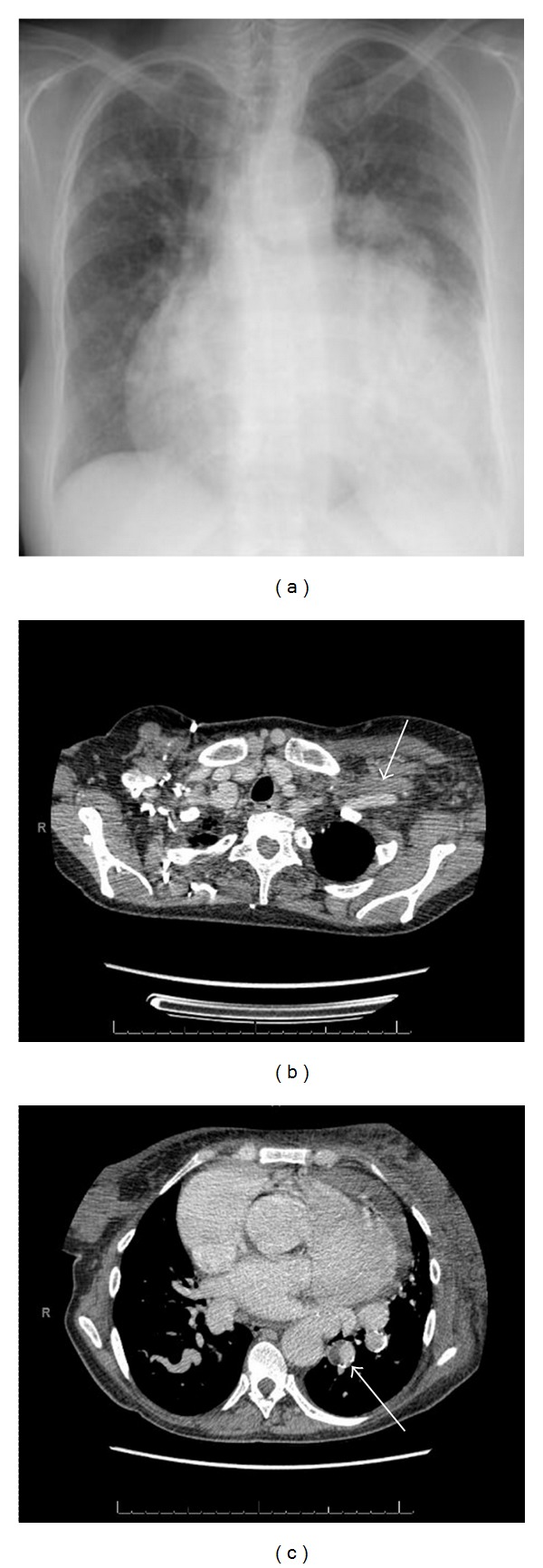# Left Hemibody Swelling in an HIV-Positive Patient with Congenital Heart Disease

**DOI:** 10.1155/2012/569095

**Published:** 2012-04-17

**Authors:** Matteo Boattini, André Almeida, Rita Barata Moura, Miguel Toscano Rico

**Affiliations:** Department of Internal Medicine, St. Marta's Hospital, 1169-024 Lisbon, Portugal

## Abstract

We report the case of a 50-year-old HIV-positive woman with a congenital cyanotic heart disease who developed left axillary, subclavian, and brachiocephalic vein thrombosis and left lower aortopulmonary collateral arterial thrombosis, presenting as left hemibody swelling. We also briefly overview the literature regarding upper extremity deep vein thrombosis (UEDVT). Given the absence of other risk factors, it was our firm believe that our patient's UEDVT was due to a hypercoagulable state associated with congestive heart failure (CHF) and HIV infection.

## 1. Introduction

Cases of UEDVT are not usual even if they have become more common due to the increased utilization of central venous catheters, cardiac pacemakers, and defibrillators. This report describes a case of a secondary UEDVT, not catheter related, caused by heart failure and HIV infection.

To our knowledge, the expressive CHF due to the congenital cyanotic cardiac disease and the HIV disease stage make this case of UEDVT unique. 

## 2. Case Presentation

A 50-year-old black woman was admitted to the hospital with dyspnea and left hemibody swelling (breast, thorax and upper and lower limbs). These symptoms had started eight days before the admission and had gradually become more severe.

The patient had a severe form of a rare, heterogeneous, congenital cyanotic heart disease with pulmonary artery atresia, ventricular septal defect, and multifocal circulation with multiple aortopulmonary collaterals (type C) [1] with resulting heart failure (NYHA class III) and chronic respiratory failure with oxygen home therapy (3 L/min).

Recent transthoracic echocardiogram revealed large ventricular septal defect, ascendant aortic aneurismatic dilatation, biventricular hypertrophy, pericardial effusion, and pulmonary atresia, with left ventricular ejection fraction of 32%.

Furthermore, she was HIV-positive and had been on highly active antiretroviral therapy (HAART) for 5 years; recent examinations showed undetectable HIV viral load (HIV-VL) and CD4 cells count of 169 for cubic millimeter of blood.

There was no history of drug allergies, nor of previous central venous cannulation.

Daily medications included furosemide 40 mg, digoxin 125 mcg, acetylsalicylic acid 100 mg, lorazepam 1 mg, HAART (tenofovir 245 mg, emtricitabine 200 mg, nevirapine 200 mg). 

**Figure 1:**
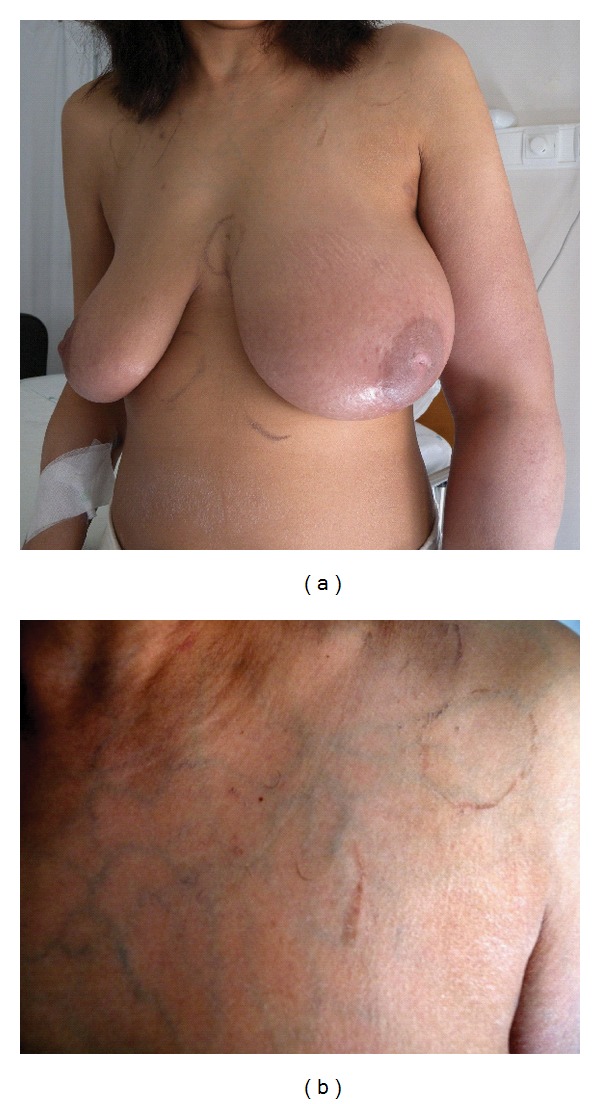


On physical examination the patient showed central cyanosis, jugular venous distention at 45 degrees, superficial venous circulation on the anterior chest wall, left hemibody swelling (particularly of the upper limb), and digital clubbing (Figures 1(a) and 1(b)).

Her body temperature was 37°C, pulse rate was 92 bpm, respiratory rate was 24/min, and blood pressure was 118/56 mmHg. 

There was no superficial lymphadenopathy. Lung examination showed decreased breath sounds over the left posterior lower chest and bibasilar rales. There was a grade III/IV loud systolic murmur heard all over the precordium and moderate hepatomegaly on abdominal examination. Blood examination showed leukocyte count 6800 × 10^3^/mcL [4500–11000], hemoglobin 13.7 g/dL [11.5–15.5], platelet count 241 × 10^3^/mcL [150–450], urea 44 mg/dL [17–43], creatinine 0.62 mg/dL [0.51–0.95], aspartate aminotransferase 28 U/L [<35], alanine aminotransferase 19 U/L [<35], gamma-glutamyltransferase 104 U/L [<38], alkaline phosphatase 116 U/L [30–120], lactate dehydrogenase 466 U/L [<247], BNP 1278 pg/mL [<100]. The hypercoagulability study was negative for deficiencies including protein S, protein C, and antithrombin III. Serum homocysteine was normal. Neither factor V Leiden mutation nor prothrombin gene mutation was detected. Anticardiolipin antibodies and lupus anticoagulant were negative too.

The electrocardiogram showed normal sinus rhythm with right bundle branch block.

The chest radiograph showed marked cardiomegaly and aortic aneurismatic dilatation (Figure 2(a)).

High-resolution computer tomography (CT) of the chest showed global cardiomegaly with pericardial effusion, thrombosis of the left axillary, subclavian and brachiocephalic veins, left chest and left upper limb edema, ascendant aortic aneurismatic dilatation (4.8 cm), descendant aortic ectasia, pulmonary atresia, small caliber of the right pulmonary artery and mural thrombosis of the left lower aortopulmonary collateral.

Color Doppler Ultrasonography highlighted no compression and no variability in flow velocity of left axillary-subclavian veins and normal morphology and compressibility of lower limbs circulation.

CT of the abdomen and pelvis confirmed hepatomegaly and detected normal permeability of the caval and iliac-femoral venous sector.

The diagnosis was left axillary, subclavian and brachiocephalic veins thrombosis, left lower aortopulmonary collateral arterial thrombosis and left lower limb lymphedema.

Due to patient comorbidities and thrombosis extension, we decided not to carry out thrombolysis, angioplasty or stent placement. A treatment with low molecular weight heparin (LMWH) in full dose was started, followed by warfarin, in order to maintain INR between 2.0 and 3.0. The patient also underwent lymphatic drainage with resolution of the edema of the left hemibody after 8 weeks of treatment.

## 3. Discussion

Cases of UEDVT are approximately 10% of DVT cases and have increased due to the greater utilization of advanced invasive procedures such as placement of central venous catheters or pacemakers [2]. The most common etiologies are malignancy, cannulation of a central vein, or both. The signs and symptoms observed in these situations are similar to those described in our patient [3].

Our patient's UEDVT resulted from a hypercoagulable state due to the CHF and, possibly, to the HIV infection. CHF meets all the requirements of Virchow's triad for a prothrombotic state [4]. Poor contractibility with low cardiac output and aberrant flow through dilated cardiac chambers, blood stasis and endothelium dysfunctions all contribute to the thrombogenesis. Severe left ventricular dysfunction, NYHA class IV patients, poor inferior vena cava collapsibility, and no anticoagulant therapy are all associated with high risk for DVT [5]. HIV infection is a relevant risk factor in itself. Among these patients, the probability of developing venous thrombosis depends on the type and number of risk factors involved, including those regarding the host, the HIV disease stage and the therapy (whether HAART or other). Low CD4 count (<200/mcL) at the time of DVT and detectable HIV-VL, with or without the presence of clinical AIDS, are the risk factors with the strongest association with venous thrombosis. Associated protein S and protein C deficiency also contribute to the increased risk [6].

Duplex ultrasonography with compression is the gold standard imaging test for patients with suspected UEDVT. CT or Magnetic Resonance Angiography may be useful for detecting underlying conditions, including anatomical abnormalities, neoplasias or lymphoadenopathies at the venous thoracic outlet [2]. In our patient the contrast-enhanced CT documented a complete filling defect of the axillary, subclavian, and brachiocephalic veins (Figure 2(b)) and a partial filling defect of the left lower aortopulmonary collateral (Figure 2(c)).

Data about medical management of UEDVT is lacking and information from lower limb thrombosis trials are used to guide the therapy. Initial anticoagulation with LMWH or unfractioned heparin followed by vitamin K antagonist during 3–6 months is considered the best choice [2].

In our patient we decided to maintain lifelong warfarin therapy because of persisting high risk of thrombosis.

## Figures and Tables

**Figure 2:**